# Osteoblastic Metastases Mimickers on Contrast Enhanced CT

**DOI:** 10.1155/2017/7278016

**Published:** 2017-07-26

**Authors:** Fahad Al-Lhedan, Sam Samaan, Wanzhen Zeng

**Affiliations:** ^1^Medical Imaging Department, King Abdullah bin Abdulaziz University Hospital, Riyadh, Saudi Arabia; ^2^Department of Nuclear Medicine, The Ottawa Hospital, Ottawa, ON, Canada

## Abstract

Secondary osseous involvement in lymphoma is more common compared to primary bone lymphoma. The finding of osseous lesion can be incidentally discovered during the course of the disease. However, osseous metastases are infrequently silent. Detection of osseous metastases is crucial for accurate staging and optimal treatment planning of lymphoma. The aim of imaging is to identify the presence and extent of osseous disease and to assess for possible complications such as pathological fracture of the load-bearing bones and cord compression if the lesion is spinal. We are presenting two patients with treated lymphoma who were in complete remission. On routine follow-up contrast enhanced CT, there were new osteoblastic lesions in the spine worrisome for metastases. Additional studies were performed for further evaluation of both of them which did not demonstrate any corresponding suspicious osseous lesion. The patients have a prior history of chronic venous occlusive thrombosis that resulted in collaterals formation. Contrast enhancement of the vertebral body marrow secondary to collaterals formation and venous flow through the vertebral venous plexus can mimic the appearance of spinal osteoblastic metastases.

## 1. Background

Approximately 16% of lymphoma patients will eventually have osseous involvement [1]. Osseous manifestations of lymphoma in the spine commonly occur as a result of direct invasion from adjacent lymph nodes; however, hematogenous osseous metastases are also a possibility [2]. The osseous involvement usually occurs during the course of the disease rather than at the initial presentation. Multiple osseous metastases are more common than a solitary metastasis. Most lymphomatous metastases tend to be osteolytic; however, osteoblastic and mixed metastases may also be encountered [2].

Osseous metastases can cause significant morbidity as a result of pathologic fracture and spinal cord compression [3, 4].

## 2. Discussion

### 2.1. Case  1

**Figure 1:**
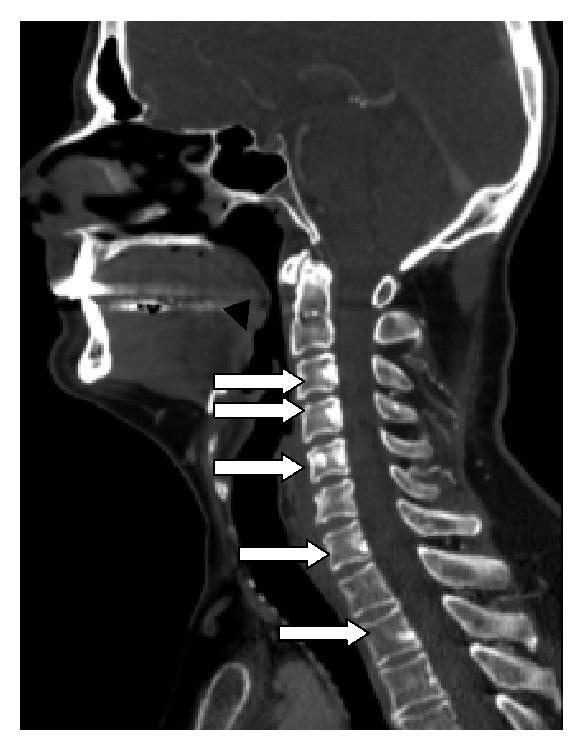
*(Neck contrast enhanced CT)*: Sagittal bone window CT demonstrating several osteoblastic lesions at C3, C4, C5, C7, and T2 (white arrows).

**Figure 2:**
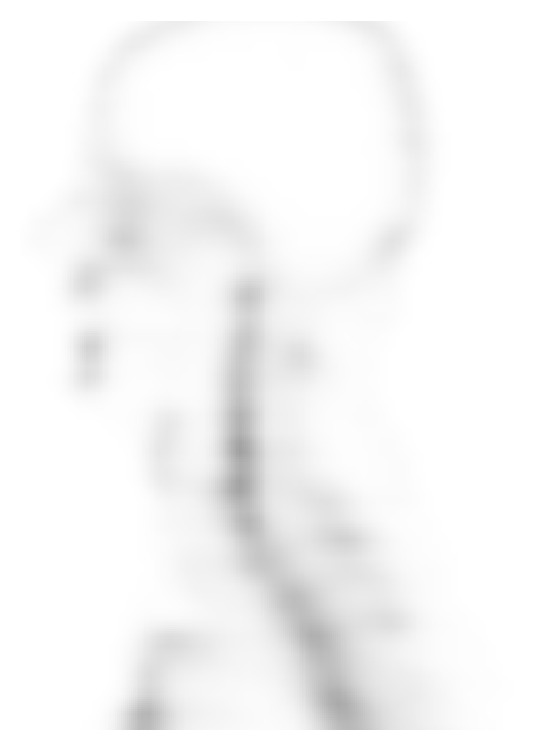
*(SPECT)*: Sagittal SPECT bone scan does not demonstrate any abnormal focal uptake within the cervical or thoracic spine.

**Figure 3:**
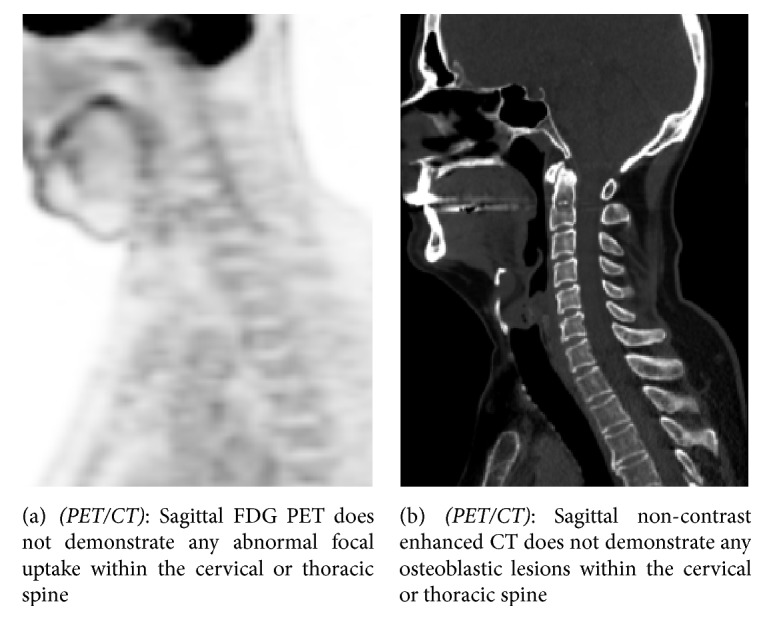


A 54-year-old male with Hodgkin lymphoma, who was in complete remission, had several new osteoblastic spinal lesions on routine follow-up contrast enhanced CT of the neck (Figure 1) which were worrisome for new osteoblastic metastases. A whole-body SPECT bone scan was performed to evaluate the extent of osseous disease (Figure 2). In addition, FDG PET/CT was performed to evaluate the disease extent within the body (Figures 3(a) and 3(b)).

The patient has a history of left innominate and left subclavian veins chronic occlusive thrombosis in addition to a partially occlusive thrombosis of the left internal jugular and left axillary veins. The routine follow-up contrast enhanced CT of the neck demonstrated at least partial occlusion of the proximal left subclavian vein with numerous collaterals in the left shoulder and left upper back region.

The lack of uptake on bone scan and FGD PET along with disappearance of lesions on the non-contrast enhanced CT portion of the PET/CT (Figure 3(b)) implies that the apparent osteoblastic lesions were merely vertebral marrow enhancement secondary to collaterals formation and venous flow through the vertebral venous plexus in the cervical and thoracic spine [5].

### 2.2. Case  2

**Figure 4:**
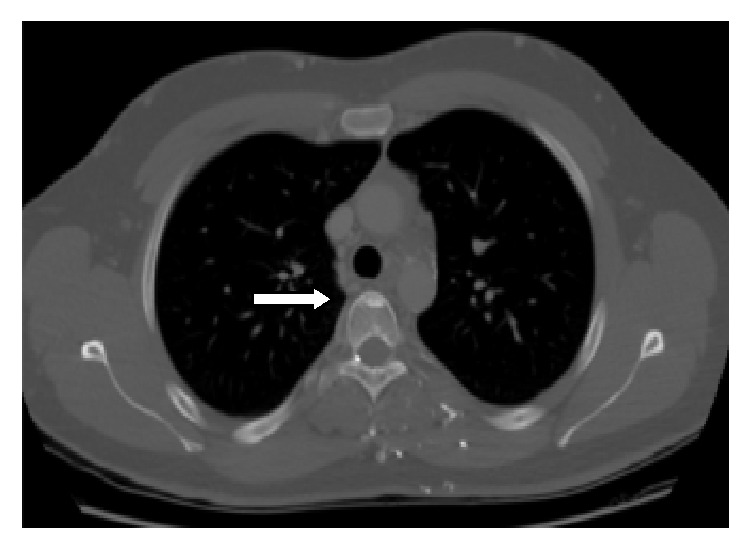
*(Chest contrast enhanced CT)*: Axial bone window CT demonstrating a solitary osteoblastic lesion at T5 (white arrow).

**Figure 5:**
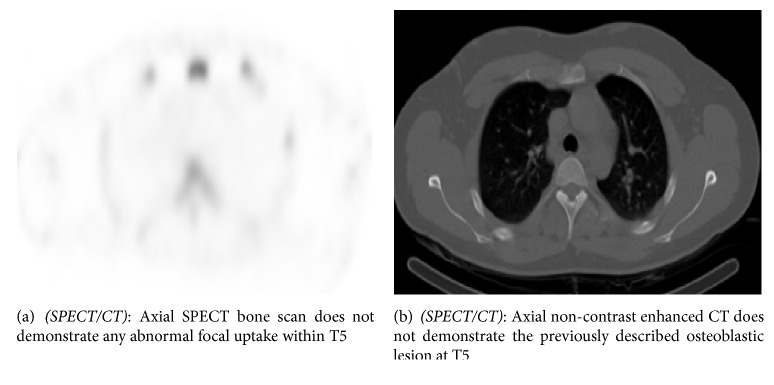


A 22-year-old male with non-Hodgkin lymphoma, who was in complete remission, had a new osteoblastic lesion at the T5 vertebral body on routine follow-up contrast enhanced CT of the chest (Figure 4) which was worrisome for a new solitary osteoblastic metastasis. A whole-body SPECT bone scan was performed to evaluate the extent of osseous disease (Figure 5).

The patient has a history of chronic occlusive thrombosis of bilateral brachiocephalic and left subclavian veins which was demonstrated on contrast enhanced CT of the chest.

Again the lack of uptake on bone scan along with disappearance of lesion on the non-contrast enhanced CT portion of the SPECT/CT (Figure 5(b)) implies that the apparent osteoblastic lesion was merely vertebral marrow enhancement secondary to collaterals formation and venous flow through the vertebral venous plexus in the thoracic spine [5].

## 3. Conclusion

The apparent osteoblastic lesions on both contrast enhanced CTs were merely marrow enhancement of the vertebral bodies. In the presence of chronic subclavian vein occlusion, collaterals often form and may result in venous flow through the vertebral venous plexus after contrast administration.

Contrast enhancement of the vertebral body marrow secondary to venous flow through the vertebral venous plexus can mimic the appearance of spinal osteoblastic metastases. Therefore, focal contrast enhancement of a vertebral body should be considered as a mimicker of an osteoblastic lesion on contrast enhanced CT in the presence of significant chronic venous occlusion and collaterals formation.

## Additional Points

*Objective*. Identify mimickers of osteoblastic metastases on contrast enhanced CT.
